# DISCRIMINATION, SOCIAL RELATIONSHIPS, AND ACCELERATED AGING AMONG MIDDLE-AGED AND OLDER AFRICAN AMERICANS

**DOI:** 10.1093/geroni/igae098.0059

**Published:** 2024-12-31

**Authors:** Karen Lincoln, Bryan Gaines, Hawa Mariko, Kaushik Golithadka, Steve Cole

**Affiliations:** University of California Irvine, Irvine, California, United States; University of California Irvine, Irvine, California, United States; University of California Irvine, Irvine, California, United States; University of Southern California, Los Angeles, California, United States; University of California Los Angeles, Los Angeles, California, United States

## Abstract

This study addresses the limited exploration of the interplay between racial discrimination, social relationships and cellular aging among African Americans. Utilizing latent class analysis, distinct risk and protective profiles were defined by perceptions of discrimination, social support, and negative interactions among middle-aged and older African Americans. Findings revealed four distinct profiles, demonstrating significant heterogeneity in experiences of discrimination, social relationships, and susceptibility to molecular stress among African Americans. The largest profile (32.09%) was characterized by high everyday and major discrimination, low family and friend support, and minimal strain. The second profile (25.37%) had wide variation, including moderate everyday discrimination, high major discrimination, very low family support, moderate friend support, moderate family and high friend strain. The smallest profile (19.4%) was characterized by high everyday and major discrimination, high family support, moderate friend support, and high strain from family and friends. Respondents in this profile had significantly elevated molecular stress compared to the reference profile (23.13%), which was characterized by low discrimination, high support, and high strain from family and friends. Regression analysis revealed that African Americans with more health conditions were more likely to belong to the high-risk profile than the reference profile. This study identified a subgroup of African Americans with an elevated risk of accelerated aging, marked by substantial racial discrimination and ambivalent social relationships. Findings provide insights into health disparities by identifying genes activated in response to discrimination and the potential influence of social factors in mitigating or exacerbating the effects of racial discrimination on cellular aging.

